# Spatio-Temporal Steering for Testing Nonclassical Correlations in Quantum Networks

**DOI:** 10.1038/s41598-017-03789-4

**Published:** 2017-06-16

**Authors:** Shin-Liang Chen, Neill Lambert, Che-Ming Li, Guang-Yin Chen, Yueh-Nan Chen, Adam Miranowicz, Franco Nori

**Affiliations:** 1 Department of Physics, National Cheng Kung University, 701 Tainan, Taiwan; 2 Naturwissenschaftlich-Technische Fakultät, Universität Siegen, 57068 Siegen, Germany; 3 CEMS, RIKEN, 351-0198 Wako-shi, Japan; 4 Department of Engineering Science, National Cheng Kung University, 701 Tainan, Taiwan; 5 Department of Physics, National Chung Hsing University, 402 Taichung, Taiwan; 6 Physics Division, National Center for Theoretical Sciences, 300 Hsinchu, Taiwan; 7 Faculty of Physics, Adam Mickiewicz University, 61-614 Poznań, Poland; 8 Department of Physics, The University of Michigan, Ann Arbor, Michigan, 48109-1040 USA

## Abstract

We introduce the concept of spatio-temporal steering (STS), which reduces, in special cases, to Einstein-Podolsky-Rosen steering and the recently-introduced temporal steering. We describe two measures of this effect referred to as the STS weight and robustness. We suggest that these STS measures enable a new way to assess nonclassical correlations in an open quantum network, such as quantum transport through nano-structures or excitation transfer in a complex biological system. As one of our examples, we apply STS to check nonclassical correlations among sites in a photosynthetic pigment-protein complex in the Fenna-Matthews-Olson model.

## Introduction

Quantum steering is an intriguing quantum phenomenon, which enables one party (usually referred to as Alice) to use her different measurement settings to remotely prepare the set of quantum states of another spatially-separated party (say Bob). This ability, which is not achievable without quantum resources, was first described by Schrödinger^1^ in his response to the work of Einstein, Podolsky, and Rosen (EPR)^2^ on quantum entanglement and the related question about the completeness of quantum mechanics. As recently shown^3^, quantum steering (also refereed to as EPR steering) is, in general, weaker than Bell’s nonlocality^4, 5^ but stronger than quantum entanglement^6^. After eighty years, quantum steering has been gradually formulated mathematically^3, 7–10^ and observed experimentally^7, 11–20^. Other developments include: using steering as a resource for quantum-information processing, quantifying steering^9, 10, 21–23^, clarifying its relationship to the problem of the incompatibility of measurements^24–28^, connecting steering with quantum computation^29, 30^, and multipartite quantum steering^30–34^, among various other generalizations and applications (see ref. 35 and references therein).

Nonclassical temporal correlations (like photon antibunching) play a fundamental role in quantum optics research, since the Hanbury-Brown and Twiss experiments^36^ and the Glauber theory of quantum coherence^37^. While there is as yet no clear temporal analog of quantum entanglement, attempts at defining such have led to new ideas about quantum causality (see, e.g., refs 38–40 and references therein). Recently, temporal steering^41^ was introduced as a temporal analog of EPR steering, which refers to a nonclassical correlation of a single object at different times. Contrary to temporal entanglement, temporal steering has a clear operational meaning^29, 41–47^. In particular, temporal steering was used for testing the security of quantum key distribution protocols^41, 46^ and for quantifying the non-Markovian dynamics of open systems^44^. Recently, temporal steering was also experimentally demonstrated^47^ by measuring the violation of the temporal inequality presented in ref. 41. Moreover, a measure of temporal steering was proposed^44, 46^ and experimentally determined^47^.

Here, we introduce the concept of spatio-temporal steering (STS) as a natural unification of the EPR and temporal forms of steering. In addition, we propose two measures of STS, specifically, its robustness and weight. We also show the usefulness of STS in testing and quantifying nonclassical correlations of quantum networks by analyzing two examples, including the decay of nonclassical correlations in quantum excitation transfers in the Fenna-Matthews-Olson (FMO) protein complex, which is one of the most widely studied photosynthetic complexes^48^. Note that STS can also be applied to test quantum-state transfer in quantum networks like those described in refs 49, 50.

## Results

### Temporal steering: From temporal hidden-variable model to temporal hidden-state model

Let us briefly review the so-called temporal hidden-state model for a single system at two moments of time^29, 41, 44^. Consider that, during the evolution of the system from time 0 to time *t*, one can perform measurements using different settings {*x*} and {*y*} to obtain outcomes {*a*} and {*b*} at times 0 and *t*, respectively. If one makes two assumptions: (A1) noninvasive measurability at time 0, which means that one can obtain a measurement outcome without disturbing the system, and (A2) macrorealism (macroscopic realism)^51^, which means that the outcome of the system pre-exists, no matter if a measurement has been performed or not. Under these conditions, there exist some hidden variables *λ*, which *a priori* determine the joint probability distributions^52–57^

1 $$p(a,b|x,y)=\sum _{\lambda }\,p(\lambda )p(a|x,\lambda )p(b|y,\lambda ).$$

Now, if one replaces the assumption (A2) with (A2’), which means that during each moment of time the system can be described by a quantum state *σ*_*λ*_, which is determined by some hidden variables *λ* independent of the measurements performed before, then the hidden variables determine not only the observed data table $p(a|x)={\sum }_{\lambda }\,p(\lambda )p(a|x,\lambda )$ at time *t* = 0, but also *a priori* the quantum state $\rho ={\sum }_{\lambda }\,p(\lambda ){\sigma }_{\lambda }$ at time *t*. It is convenient to define the *temporal assemblage*

$${\{{\sigma }_{a|x}^{{\rm{T}}}(t)\equiv p(a|x){\tilde{\sigma }}_{a|x}(t)\}}_{a,x},$$

where ${\tilde{\sigma }}_{a|x}(t)$ is the observed quantum state at time *t* conditioned on the earlier measurement event *a*|*x* at time 0. Thus, the temporal assemblage is a set of subnormalized states, which characterizes the *joint behaviour*: (1) $p(a|x)={\rm{tr}}[{\sigma }_{a|x}^{{\rm{T}}}(t)]$ and (2) ${\tilde{\sigma }}_{a|x}(t)={\sigma }_{a|x}^{{\rm{T}}}(t)/{\rm{tr}}[{\sigma }_{a|x}^{{\rm{T}}}(t)]$. Furthermore, the formulation of the temporal hidden-state model can be written as

2 $${\sigma }_{a|x}^{{\rm{T}}}(t)=\sum _{\lambda }\,p(\lambda )p(a|x,\lambda ){\sigma }_{\lambda }.$$

Quantum mechanics predicts some assemblages, which do not admit the temporal hidden-state model, and we refer to this situation as *temporal steering* ^44^. Note that since the hidden-state model is a strict subset of the hidden-variable model, using the former model may admit an easier detection of the nonclassicality of the quantum dynamics than using the hidden-variable model.

### Spatio-temporal steering

**Figure 1:**
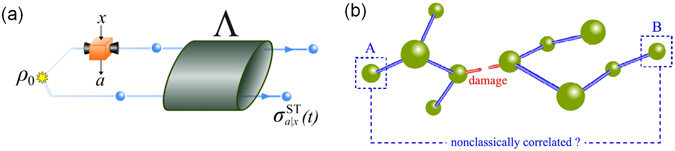
(**a**) Schematic diagram of spatio-temporal steering. At time *t* = 0, a system A (which may be entangled with a system B) is subject to a local measurement with one of the measurement settings {*x*}, which is described by a positive-operator-valued measure {*F*_*a*|*x*_}_*a*_. After this measurement, the post-measurement composite state *ρ*_*a*|*x*_ is sent into a quantum channel Λ and evolves for a time period *t*. After many rounds of the experiment, the set of subnormalized quantum states of the system B is denoted as ${\{{\sigma }_{a|x}^{{\rm{ST}}}(t)\}}_{a,x}$. With some appropriate *ρ*_0_, {*F*_*a*|*x*_}_*a*,*x*_, and Λ, the assemblage ${\{{\sigma }_{a|x}^{{\rm{ST}}}(t)\}}_{a,x}$ does not admit the spatio-temporal hidden-state model equation (3). We call this *spatio*-*temporal steering* and refer the assemblage ${\{{\sigma }_{a|x}^{{\rm{ST}}}(t)\}}_{a,x}$ as *spatio*-*temporal steerable*. (**b**) A schematic example of a quantum network with damage (strong dissipation or dephasing, or an entirely broken link). The STS weight and robustness can be employed as diagnostic tools to check whether site-A and site-B are nonclassically correlated.

Similarly, we can also generalize the hidden-state model to the hybrid spatio and temporal scenario. That is, we would like to consider the hidden-state model for a system B at time *t*, after the local measurement has been performed on a system A at time 0. Then, under the assumptions of non-invasive measurement for the system A at time 0 and the hidden state for the system B at time *t*, the spatio-temporal hidden-state model is written as (for brevity, the term “spatio-temporal” will be sometimes omitted hereafter).

3 $${\sigma }_{a|x}^{{\rm{ST}},{\rm{B}}}(t)=\sum _{\lambda }\,p(\lambda ){p}_{{\rm{A}}}(a|x,\lambda ){\sigma }_{\lambda }^{{\rm{B}}}\quad \forall \,a,x,$$

where ${\sigma }_{a|x}^{\mathrm{ST},B}(t)\equiv {p}_{{\rm{A}}}(a|x){\tilde{\sigma }}_{a|x}^{{\rm{ST}},{\rm{B}}}(t)$, with ${\tilde{\sigma }}_{a|x}^{{\rm{ST}},{\rm{B}}}(t)$ being the observed quantum state of the system B at time *t*, conditioned on the measurement event *a*|*x* [with corresponding data table *p*_A_(*a*|*x*)] of the system A at time 0. When there is no risk of confusion, we will abbreviate ${\sigma }_{a|x}^{{\rm{ST}},{\rm{B}}}(t)$ as ${\sigma }_{a|x}^{{\rm{ST}}}(t)$, *p*_A_(*a*|*x*) as *p*(*a*|*x*), and ${\sigma }_{\lambda }^{{\rm{B}}}$ as *σ*_*λ*_. The set of subnormalized states ${\{{\sigma }_{a|x}^{{\rm{ST}}}(t)\}}_{a,x}$ is refereed to as a *spatio*-*temporal assemblage* having the property $p(a|x)={\rm{tr}}[{\sigma }_{a|x}^{{\rm{ST}}}(t)]$ and ${\tilde{\sigma }}_{a|x}^{{\rm{ST}}}(t)={\sigma }_{a|x}^{{\rm{ST}}}(t)/{\rm{tr}}[{\sigma }_{a|x}^{{\rm{ST}}}(t)]$, and can be certified if it admits the model, given by equation (3), via the following semidefinite programming (SDP) (see ref. 58 for SDP, and refs 8, 9, 27 for dealing with the certification of the hidden-state model for a given assemblage):

4 $$\begin{array}{cccc}\quad \quad \,{\rm{f}}{\rm{i}}{\rm{n}}{\rm{d}}\,\,\{{\rho }_{\lambda }\} &  &  & \\ {\rm{s}}{\rm{u}}{\rm{b}}{\rm{j}}{\rm{e}}{\rm{c}}{\rm{t}}\,{\rm{t}}{\rm{o}}\,\,\,{\sigma }_{a|x}^{{\rm{S}}{\rm{T}}}(t) & = & {\sum }_{\lambda }\,p(a|x,\lambda ){\rho }_{\lambda } & {\rm{\forall }}\,a,x,\\ \quad \quad \quad \,\,\,\,\,{\rm{t}}{\rm{r}}{\sum }_{\lambda }\,{\rho }_{\lambda } & = & 1,\quad \quad \quad \,\,{\rho }_{\lambda }\ge 0 & {\rm{\forall }}\,\lambda ,\end{array}$$

where ${\rho }_{\lambda }\equiv p(\lambda ){\sigma }_{\lambda }$, and the notation *ρ*_*λ*_ ≥ 0 denotes that *ρ*_*λ*_ is a positive-semidefinite operator. Quantum mechanics predicts that

$${\sigma }_{a|x}^{{\rm{ST}}}(t)={{\rm{tr}}}_{{\rm{A}}}\{{\rm{\Lambda }}[(\sqrt{{F}_{a|x}}\otimes {\mathbb{1}}){\rho }_{0}(\sqrt{{F}_{a|x}}\otimes {\mathbb{1}})]\},$$

with *ρ*_0_ being the initial quantum state shared by the systems A and B at time 0, {*F*_*a*|*x*_}_*a*_ being the positive-operator-valued measure representing the measurement *x*. The quantum channel Λ describes the time evolution of the post-measurement composite system from time 0 to time *t* see the schematic diagram in Fig. 1(a).

With an appropriately designed *ρ*_0_, {*F*_*a*|*x*_}_*a*,*x*_, and Λ, the assemblage cannot be written in the form of equation (3) i.e., there is no feasible solution of the SDP problem given in equation (4). In this situation, the assemblage is said to be *spatio*-*temporal steerable*. To quantify the degree of such steerability, we would like to introduce the quantifier called the *STS weight* (*ST* *SW*), which is defined as

$$STSW=\,min\,(1-\mu )\,{\rm{s}}{\rm{u}}{\rm{b}}{\rm{j}}{\rm{e}}{\rm{c}}{\rm{t}}\,{\rm{t}}{\rm{o}}\,{\{{\sigma }_{a|x}^{{\rm{S}}{\rm{T}}}(t)=\mu {\sigma }_{a|x}^{{\rm{S}}{\rm{T}},{\rm{U}}{\rm{S}}}(t)+(1-\mu ){\sigma }_{a|x}^{{\rm{S}}{\rm{T}},S}(t)\}}_{a,x}$$

(the same techniques have been demonstrated in refs 9, 44). ${\{{\sigma }_{a|x}^{\mathrm{ST},\mathrm{US}}(t)\}}_{a,x}$ stands for the unsteerable (US) assemblage i.e., one admits equation (3), ${\{{\sigma }_{a|x}^{\mathrm{ST},S}(t)\}}_{a,x}$ represents the steerable assemblage, and 0 ≤ *μ* ≤ 1. This can be formulated as the following SDP problem:

5 $$\begin{array}{cc}STSW=\,min\,\,(1-{\rm{t}}{\rm{r}}{\sum }_{\lambda }\,{\rho }_{\lambda }),\quad {\rm{w}}{\rm{i}}{\rm{t}}{\rm{h}}\quad {\rho }_{\lambda }\ge 0 & {\rm{\forall }}\,\lambda \\ \quad {\rm{s}}{\rm{u}}{\rm{b}}{\rm{j}}{\rm{e}}{\rm{c}}{\rm{t}}\,\,{\rm{t}}{\rm{o}}\,\,\,{\sigma }_{a|x}^{{\rm{S}}{\rm{T}}}(t)-{\sum }_{\lambda }\,p(a|x,\lambda ){\rho }_{\lambda }\ge 0 & {\rm{\forall }}\,a,x.\end{array}$$

In addition, we would like to introduce another measure, referred to as the *STS robustness* (*ST* *SR*), which can be viewed as a generalization of the EPR steering robustness^10^ to the present spatio-temporal scenario. The STS robustness *ST* *SR* can be defined as the minimum noise ${\tau }_{a|x}^{{\rm{ST}}}(t)$ to be added to ${\sigma }_{a|x}^{{\rm{ST}}}(t)$, such that the mixed assemblage is unsteerable. That is, $ST\,SR=\,{\rm{\min }}\,\alpha \,{\rm{subject}}\,{\rm{to}}\,{\{\frac{1}{1+\alpha }{\sigma }_{a|x}^{{\rm{ST}}}(t)+\frac{\alpha }{1+\alpha }{\tau }_{a|x}^{{\rm{ST}}}(t)={\sigma }_{a|x}^{\mathrm{ST},\mathrm{US}}\}}_{a,x}$. This can also be formulated as an SDP problem. Specifically,

6 $$\begin{array}{ll}ST\,SR=\,{\rm{\min }}\,({\rm{tr}}{\sum }_{\lambda }\,{\rho }_{\lambda }-1),\quad {\rm{with}}\quad {\rho }_{\lambda }\ge 0 & \forall \,\lambda \\ \quad {\rm{subject}}\,{\rm{to}}\,{\sum }_{\lambda }\,p(a|x,\lambda ){\rho }_{\lambda }-{\sigma }_{a|x}^{{\rm{ST}}}(t)\ge 0 & \forall \,a,x.\end{array}$$

The STS robustness and weight, analogously to their EPR counterparts, have different operational meanings and properties. For example, one could expect that these measures can imply different orderings of states, analogously to this property exhibited by various measures of entanglement^59–61^, Bell nonlocality^62^, and nonclassicality^63^. A detailed comparison of these two STS measures will be given elsewhere^64^. Here, we have calculated the STS weight for Example 1, and the STS robustness for Example 2 in the following sections, just to show that these measures can easily be computed and interpreted.

### Examining nonclassical correlations within a quantum network

A possible application of STS is that it can be used to witness whether two nodes of a quantum network are *nonclassically correlated* (or *quantum connected*). Consider two qubits on the opposite ends of a quantum network, as shown in Fig. 1(b). There may be a damage somewhere in the network, such that the quantum coherent interaction between distant nodes may be inhibited. To verify this, one can initially perform measurements at time *t*_A_ = 0 on site-A. On site-B, one performs measurements at a later time *t*. If the value of the STS weight (or, equivalently, the STS robustness) is always zero for the whole range of time *t*, one can say that the influence of the quantum measurement at site-A is not transmitted to site-B in a steerable way.

#### Example 1: The spatio-temporal steering weight in a three-qubit network

**Figure 2:**
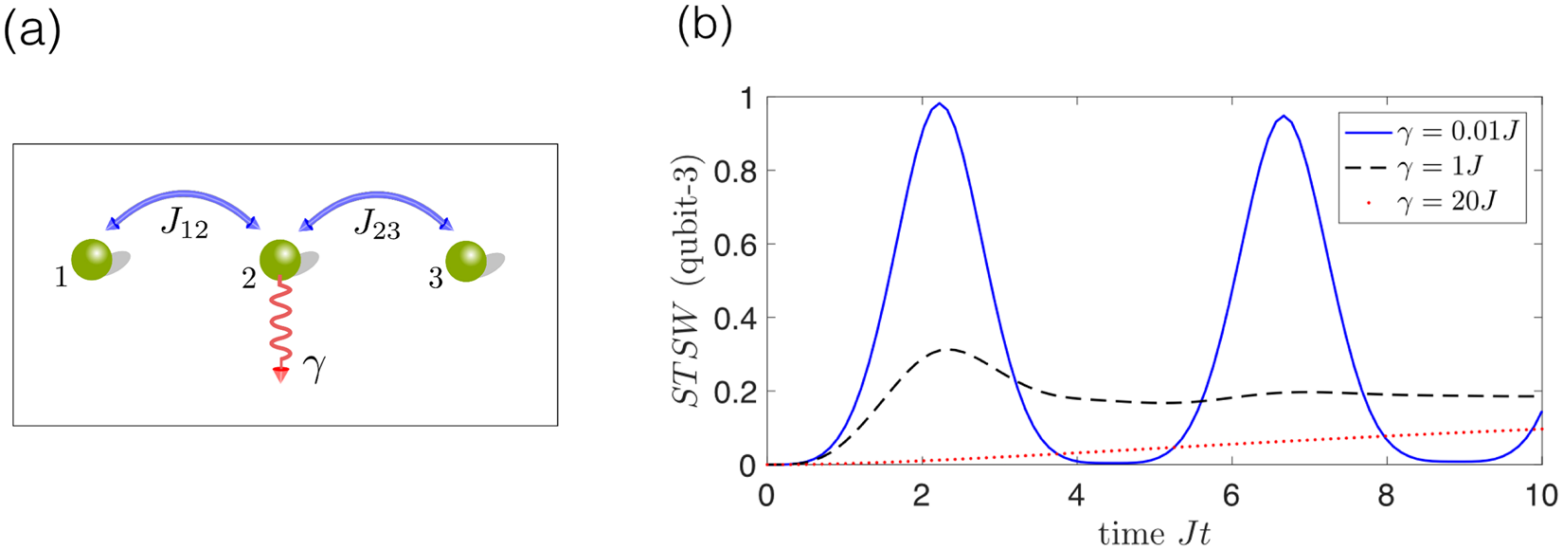
The STS weight versus time in a simple quantum network model described in example 1 in the text. (**a**) Three identical qubits, with coherent coupling *J*_12_ (*J*_23_) between qubit 1 (2) and 2 (3). To simulate the damaged node, we assume qubit 2 suffers a phase damping *γ*. (**b**) The blue-solid, black-dashed, and red-dotted curves show the STS weight (*ST* *SW*) of the assemblage ${\{{\sigma }_{a|x}^{{\rm{ST}}}(t)\}}_{a,x}$ of qubit-3 for different dephasing rates of the middle qubit *γ*/*J* = 0.01, 1, and 20, respectively. The measurement settings {*x*} on qubit-1 at time 0 are the Pauli set *X*, *Y*, and *Z*. The initial condition is $|1\rangle \otimes |0\rangle \otimes |0\rangle$, and ${J}_{12}={J}_{23}\equiv J$. The time *t* is in units of *J* ^−1^. From the figure, we can see that when the dephasing rate increases from 0.01*J* to 1*J*, the amplitude of the *ST* *SW* decreases. This means that when dephasing rate increases from 1*J* to 20*J*, the dephasing mechanism dominates the dynamics of the system, leading to a disappearance of the oscillatory behavior. Although the dephasing rate is large (e.g., the red-dotted curve), the effect of the measurement on qubit-1 at time 0 can still be transited to qubit-3 via the coherent coupling between the qubits, making the *ST* *SW* gradually increase. For brevity, we are omitting analogous plots for the STS robustness.

As an example of STS in a quantum network, let us apply a simplified model of two qubits coherently coupled via a third qubit Fig. 2(b). The interaction Hamiltonian of the entire system is

7 $${H}_{{\rm{int}}}=\hslash {J}_{12}({\sigma }_{+}^{1}{\sigma }_{-}^{2}+{\sigma }_{-}^{1}{\sigma }_{+}^{2})+\hslash {J}_{23}({\sigma }_{+}^{2}{\sigma }_{-}^{3}+{\sigma }_{-}^{2}{\sigma }_{+}^{3}),$$

where ${\sigma }_{+}^{i}$ (${\sigma }_{-}^{i}$) is the raising (lowering) operator of the *i*th qubit respectively, while *J*_12_ (*J*_23_) is the coupling strength between qubits 1 (2) and 2 (3). To simulate the damage in the network, and quantify it, we assume qubit 2 may suffer noise-induced dephasing. For simplicity, the two coupling strengths are equal, i.e., ${J}_{12}={J}_{23}\equiv J$. The STS weight, calculated as described above, is plotted in Fig. 2(b). We can see that if the dephasing rate *γ* is very small, the STS weight oscillates with time *t*, revealing the coherent interaction between qubits 1 and 3 via the middle qubit. If *γ* is large (i.e. the middle node is damaged), one sees the growth of the STS weight at a later time. One can imagine that if the dephasing is very strong, it can inhibit the appearance of the STS weight. However, several caveats arise in that the apparent correlations may be transmitted via other means than the network itself (via some environment or eavesdropper). A possible opening for future research in this area is to consider a multi-partite extension, and whether it can be used as a measure of quantum communities in networks^65^.

#### Example 2: The spatio-temporal steering robustness in the Fenna-Matthews-Olson complex

**Figure 3:**
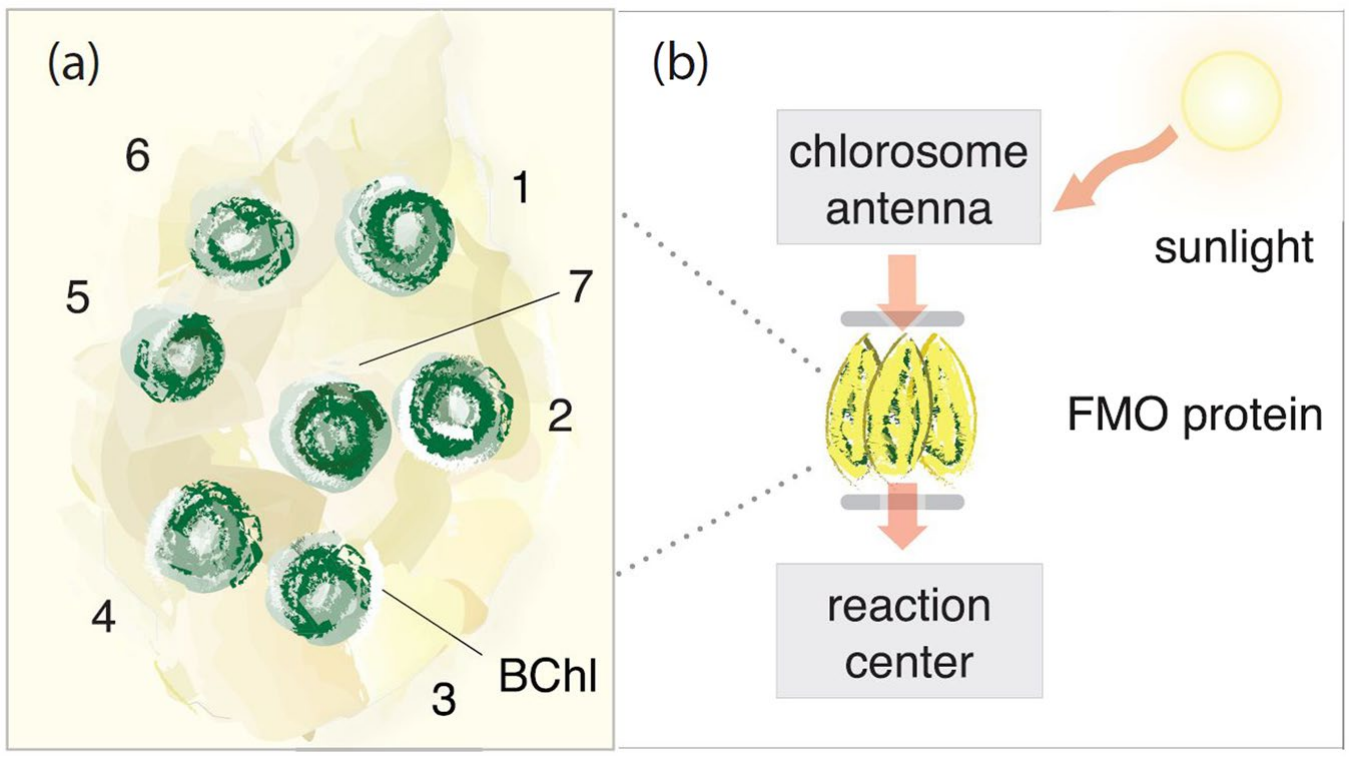
(**a**) Schematic diagram of a single monomer of the FMO protein complex. This monomer contains eight sites (here we show only seven of them). In the bacterial photosynthesis, the excitation from the light-harvesting antenna enters the FMO complex at sites 6 or 1 and is then transferred from one site to another. The excitation can irreversibly jump to the reaction center, when it reaches site-3. In this work, the initial condition is set as site-6 in a mixed excited state while the other sites are in ground states. BChl stands for a bacteriochlorophyll molecule. (**b**) Schematics of how the monomer exists in a trimer, and acts as a wire connecting a large antenna complex to the reaction center.

Much attention has been devoted to the possible functional role of quantum coherence^66, 67^ in photosynthesis bacteria, since the observation of possible quantum coherent motion of an excitation within the FMO complex – a photosynthetic pigment-protein complex^68–70^. A simple treatment of the excitation transfer in the FMO complex normally considers seven coupled sites (chromophores), as shown in Fig. 3, and their interaction with the environment. The hierarchy method^71–75^ or other open-quantum system models^76, 77^ can be used to explain the presence of quantum coherence and predict the physical quantities observed in experiments.

Empowered by STS, one can ask the following questions for a network like the FMO protein complex: When an excitation arrives at site-6, and propagates through the network, how large is its quantum influence, if any, to other sites? When do such nonclassical correlations vanish? Previously, quantum entanglement in the FMO complex has been theoretically analyzed^78^. Given the fact that the excitation transfer is *dynamic* in nature, with a specific starting site (site-1 or site-6), it is more natural to examine the nonclassical correlation between sites at different times by using the STS measures. However, we point out that evaluating these measures requires measurements in different “excitation” bases at both source and target sites. Thus, evaluating these measures represents an analysis of the network itself, and how quantum correlations propagate through it, much akin to the approach taken in ref. 79.

The model Hamiltonian of the single FMO monomer containing *N* sites can be written as (see, e.g. ref. 80 and references therein):

8 $$H=\sum _{n=1}^{N}\frac{{\varepsilon }_{n}}{2}{\sigma }_{z}^{(n)}+\sum _{n < n^{\prime} }\,{J}_{n,n^{\prime} }({\sigma }_{+}^{(n)}{\sigma }_{-}^{(n^{\prime} )}+{\sigma }_{-}^{(n)}{\sigma }_{+}^{(n^{\prime} )})$$

where the state Pauli operators represent an electronic excitation at site *n*, (*n* $\in$ 1, …, 7), such that ${\sigma }_{z}^{(n)}=|{e}^{(n)}\rangle \langle {e}^{(n)}|-|{g}^{(n)}\rangle \langle {g}^{(n)}|$, *ε*_*n*_ is the site energy of chromophore *n*, and *J*_*n*,*n*′_ is the excitonic coupling between the *n*th and *n*′th sites. In the literature, because of the rapid recombination of multiple excitations in such a complex, it is common to simplify drastically this model by assuming that the whole complex only contains a single excitation. In that case the 2^7^ dimensional Hilbert space is reduced to a 7 dimensional Hilbert space. Here, while we also assume only a single-excitation, we keep the full 2^7^ dimensional Hilbert space to enable us to consider measurements in a basis which represent superpositions of excitations at various sites. (Note that for simplicity, we omit the recently discovered eighth site^81^).

In the regime that the excitonic coupling *J*_*n*,*n*′_ is large compared with the reorganization energy, the electron-nuclear coupling can be treated perturbatively^82^, and the open-system dynamics of the system can be described by the Haken-Strobl master-type equation^83, 84^,

9 $$\dot{\rho }(t)=-\frac{i}{\hslash }[H,\rho ]+L[\rho ],$$

where *ρ* is the system density matrix, and *L*[*ρ*] denotes the Lindblad operators

10 $$L[\rho ]={L}_{{\rm{sink}}}[\rho ]+{L}_{{\rm{deph}}}[\rho ],$$

where the Lindblad superoperator *L*_sink_ describes the irreversible excitation transfer from site-3 to the reaction center:

11 $${L}_{{\rm{sink}}}[\rho ]={\rm{\Gamma }}[2s\rho {s}^{\dagger }-{s}^{\dagger }s\rho -\rho {s}^{\dagger }s],$$

where $s={\sigma }_{+}^{(R)}{\sigma }_{-}^{\mathrm{(3)}}$, with ${\sigma }_{+}^{(R)}$ representing the creation of an excitation in the reaction center, and Γ denotes the transfer rate. The other Lindblad superoperator, *L*_deph_, describes the temperature-dependent dephasing with the rate *γ*_dp_:

12 $${L}_{{\rm{deph}}}[\rho ]={\gamma }_{{\rm{dp}}}\sum _{n}\,[2{A}_{n}\rho {A}_{n}^{\dagger }-{A}_{n}{A}_{n}^{\dagger }\rho -\rho {A}_{n}{A}_{n}^{\dagger }],$$

where ${A}_{n}={\sigma }_{z}^{(n)}$. This dephasing Lindblad operator leads to the exponential decay of the coherences between different sites in the system density matrix. The pure-dephasing rate *γ*_dp_ can be estimated by applying the standard Born-Markov system-reservoir model^85, 86^. We assume an Ohmic spectral density, which, combined with the Born-Markov approximations, leads to a dephasing rate directly proportional to the temperature^86^. While more complex treatments are necessary to fully describe the true dynamics of the FMO complex, here we restrict ourselves to this weak-coupling Lindblad form for numerical efficiency and easier interpretation of results. Note that there exists a factor 1/8 between the dephasing rate *γ*_dp_ here and the orthodox one in the 7-site model.

In the FMO monomer, the excitation transferring from site-3 to the reaction center takes place on a time scale of ~1 ps, and the dephasing occurs on a time scale of ~100 fs^86^. These two time scales are both much faster than that of the excitonic fluorescence relaxation (~1 ns), which is, thus, omitted here for simplicity. Here we present the values used for the system Hamiltonian in calculating the excitation transfer^87^:

$$H^{\prime} =(\begin{array}{ccccccc}215 & -104.1 & 5.1 & -4.3 & 4.7 & -15.1 & -7.8\\ -104.1 & 220 & 32.6 & 7.1 & 5.4 & 8.3 & 0.8\\ 5.1 & 32.6 & 0 & -46.8 & 1.0 & -8.1 & 5.1\\ -4.3 & 7.1 & -46.8 & 125 & -70.7 & -14.7 & -61.5\\ 4.7 & 5.4 & 1.0 & -70.7 & 450 & 89.7 & -2.5\\ -15.1 & 8.3 & -8.1 & -14.7 & 89.7 & 330 & 32.7\\ -7.8 & 0.8 & 5.1 & -61.5 & -2.5 & 32.7 & 280\end{array})$$

Here the diagonal elements correspond to *ε*_*n*_, and the off-diagonals to *J*_*n*,*n*′_. We omit the large ground-state off-set, as it does not influence the results. This FMO dynamics description is based on our former work^80^.

**Figure 4:**
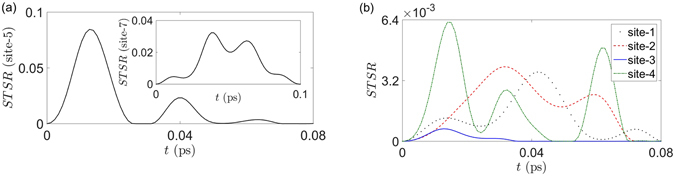
Evolution of the STS robustness (*ST* *SR*) in the FMO complex. (**a**) The main figure together with the insets show the decays of the STS robustness of the assemblages ${\{{\sigma }_{a|x}^{{\rm{ST}}}(t)\}}_{a,x}$ of site-5 and site-7 respectively. (**b**) The black-dotted, red-dashed, blue-solid, and green dash-dotted curves are represent STS robustness of the assemblages ${\{{\sigma }_{a|x}^{{\rm{ST}}}(t)\}}_{a,x}$ of site-1, 2, 3, and 4 respectively. As the previous case, the measurement settings on site-6 at time 0 are the Pauli set *X*, *Y*, and *Z*. We assumed that the FMO is cooled down to *T* = 15 K, the FMO initial state is completely mixed at site-6 while the other sites are in ground state, the dephasing rate is 7.7 cm^−1^, and the decay rate is 5.3 cm^−1^. Again, for brevity, we do not present analogous plots for the STS weight.

In Fig. 4, we numerically calculated the STS robustness of site-6 to other sites by using the Haken-Strobl equation of motion^80, 84^. In plotting this figure, the temperature is chosen to be *T* = 15 K with the corresponding dephasing rate *γ*_dp_ = 7.7 cm^−1^ and the decay rate (into the reaction center from site-3 only) Γ = 5.3 cm^−1^. As seen from this figure, the largest STS robustness occurs from site-6 to site-5. This is because site-6 and site-5 have the second largest intersite coupling (≈89.7 cm^−1^) in the whole network. Another interesting fact is that the robustness of site-6 to site-7 has the second largest magnitude (with a time delay) and the longest *vanishing time* (death time) of the STS robustness. In view of the coupling strength of the Hamiltonian, this may be due to the relative strong couplings of site-5 to site-4 (≈70.7 cm^−1^) and site-4 to site-7 (≈61.5 cm^−1^), such that the influence from site-6 is transferred through these sites with a time delay. In other words, the STS robustness not only gives the magnitude of the nonclassical correlations between two sites, but also gives the information of how long the nonclassical correlation takes to arrive, and how long it can be sustained.

## Conclusions

Although the concept of spatio-temporal quantum entanglement is fundamentally difficult to be described consistently, we showed that STS, describing a certain type of spatio-temporal nonclassical correlations, can indeed be defined and quantified in an operational way. We hope that this may provide a wider view than the purely spatial or temporal correlations separately. In addition, we showed that STS, with its measures, including the STS weight and STS robustness, can be useful to assess nonclassical correlations in quantum networks or other open quantum systems. As an application, we described two examples of testing nonclassical correlations in a toy model of a three-qubit quantum network and in a more realistic model of the excitation transfer in the seven-site FMO complex. We believe that STS can be useful also for testing nonclassical correlations of more complex biological systems^66, 79^ and for describing quantum transport through artificial nano-structures^88–91^. Finally, we mention that a possible experimental demonstration of STS can be based on a delayed-time modified version of the experiment on temporal steering reported in ref. 47.
